# A study of traumatic brain injury in Puerto Rico

**DOI:** 10.1097/ph9.0000000000000083

**Published:** 2025-06-05

**Authors:** Manuel F. Mas, Iván J. Pérez, Natasha L. Frontera, Sebastián Martínez, José G. Conde, Walter R. Frontera

**Affiliations:** a Department of Physical Medicine, Rehabilitation & Sports Medicine, University of Puerto Rico School of Medicine, San Juan, Puerto Rico; b Department of Surgery, University of Puerto Rico School of Medicine, San Juan, Puerto Rico; c University of Puerto Rico School of Medicine, San Juan, Puerto Rico; d Biomedical Sciences Graduate Program, University of Puerto Rico School of Medicine, San Juan, Puerto Rico; e Department of Physiology, University of Puerto Rico School of Medicine, San Juan, Puerto Rico

**Keywords:** Trauma, Motor vehicle collisions, Falls, Glasgow Coma Scale, Injury severity score

## Abstract

**Objective:**

Characterize the traumatic brain injury (TBI) population in the Puerto Rico Trauma Hospital.

**Materials and Methods:**

Retrospective review of data from all encounters with ICD-10-CM codes corresponding to the US National Center for Health Statistics, CDC, TBI case definition in calendar years 2019–2022. Variables included age, sex, mechanism of injury, Glasgow Coma Scale, Injury Severity Score, length of stay, and discharge disposition.

**Results:**

A total of 947 patients with TBI were identified after record review. The majority were men (80.7%), and the median age was 39 years. Most cases were severe, with a median Glasgow Coma Scale of 7 and a median Injury Severity Score of 17. The most common mechanisms of injury were motorcycle injuries (23.0%) and motor vehicle collisions (22.9%). Falls accounted for 19.9% of cases, and the occurrence increased with age. The median length of stay was 20 days with no difference between sexes (*P* = 0.24). In hospital mortality (18.1%) was similar across all calendar years and higher (*P* < 0.001) for males (20.7%) compared with females (8.2%). More females (84.6%) were discharged home than males (69.9%) (*P* < 0.001).

**Conclusions:**

TBI care is a service in high demand in Puerto Rico, with a high burden and severity of injury among young men. The next step should include a detailed characterization of TBI in other hospitals in Puerto Rico and on strengthening rehabilitation services in health systems on the island. Comprehensive preventive strategies are required to address key mechanisms of TBI in Puerto Rico.

## Introduction

Traumatic brain injury (TBI) includes a broad spectrum of pathology in the central nervous system with a large cascade of sequelae following initial injury.^[1]^ Long-term consequences of TBI may include significant functional impairments such as mobility limitations, serious cognitive and behavioral problems, and high mortality. In 2013, ~2.5 million TBI-related emergency department visits were reported in the United States.^[2]^ More recently, there were over 69,000 TBI-related deaths in the United States in 2021.^[3]^ The Global Burden of Disease Study 2021 reported 20.84 million new cases and 37.93 million prevalent cases, with an age-standardized incidence rate of 259.0 cases/100,000 individuals.^[4]^ It has been estimated that patients with TBI have 5.48 million years living with disability after injury.^[5]^ Thus, research efforts to better understand the epidemiology and nature of these injuries may help the development and implementation of health policies to reduce the occurrence and severity of TBIs and to mitigate their sequelae.

Few studies on TBI have been conducted in Puerto Rico. One study described 332 elderly cases of TBI resulting from falls at the Puerto Rico Medical Center.^[6]^ In another study, Brau et al^[7]^ reported traumatic injuries of the central nervous system evaluated in the Puerto Rico Medical Center from 2006 to 2011.^[7]^ In that study, most of the data analysis combined all neurological injuries, and details about TBI cases were not reported. A third study reported on all trauma patients admitted to the Trauma Hospital of Puerto Rico (THPR) but did not analyze TBI cases separately.^[8]^ Therefore, detailed information about TBI in Puerto Rico based on recent data is warranted, including studying the severity of the injuries and discharge disposition patterns.

The present study was performed to describe the TBI population admitted to the THPR. The specific aims were to (1) describe the patient population with TBI admitted to the THPR, (2) characterize the mechanism and severity of the injuries, and (3) describe the discharge disposition of these patients. We hypothesized that (1) the characteristics of the patient population of the THPR are similar to those described in other TBI populations; (2) there is a bimodal distribution of age with a higher number of cases among young and older adults, (3) the most frequent mechanism of injury in younger age groups is motor vehicle collisions (MVC), while TBI in older adults is mostly related to falls, and (4) the in-hospital mortality rate is high.

## Materials and Methods

The present report is part of a larger study of neurotrauma in Puerto Rico during calendar years 2019–2022.^[9]^

### Setting

The THPR is the island’s only trauma center. It is a level 2 center where the staff of THPR is composed of board-certified trauma physicians and specialists in 19 other medical domains, including neurosurgery and physical medicine and rehabilitation (PM&R). The THPR has 31 intensive care beds, 47 general beds, and 8 beds dedicated to inpatient rehabilitation, and admitted 5126 patients during the 4 calendar years included in this study. Patients with isolated damage to the brain or spine are admitted to the Neurosurgery Service of the nearby University District Hospital. Otherwise, they are admitted to the THPR. Hence, TBI patients admitted to the THPR usually have multiple traumatic injuries.

### Study design, data source, and collection

Data from admissions between January 1, 2019, and December 31, 2022, was sourced from the THPR’s NTRACS Trauma Registry (NTRS) and exported as an Excel file. This was supplemented by a review of the electronic medical records system. The study was approved by the Institutional Review Board of the University of Puerto Rico Medical Sciences Campus (Protocol # 22100030034). Initially, International Classification of Diseases, 10th Revision, Clinical Modification (ICD-10-CM) codes for injury were used to select patient encounters in the NTRS that were compatible with the US National Center for Injury Prevention and Control and the US National Center for Health Statistics preliminary TBI case definition.^[10–12]^ Thus, encounters with ICD-10-CM code groups S02.0, S02.1, S02.8, S02.91, S04.02, S04.03, S04.04, S06, S07.1, and T74.4 on admission were selected for record review. A total of 1175 unique records met the preliminary TBI case definition.

Three of the authors reviewed records individually to confirm the diagnosis of TBI. This was achieved by review of history and physical examination on admission; trauma, neurosurgery, and PM&R consults; discharge summary; and progress notes. If the patient had a documented diagnosis of TBI a second review was performed by a specialist in Brain Injury Medicine/PM&R. The final number of confirmed, unique TBI cases was 947.

Data collected for analysis included age at the time of injury, sex, mechanism of trauma, injury severity score (ISS), Glasgow Coma Scale (GCS), length of stay at the THPR, in-hospital mortality, evidence of PMR consultation (yes/no), and discharge destination. Seventy-nine cases (8.3%) were found to have data entry errors for the GCS in the NTRS. These were corrected after obtaining the original GCS values from the electronic medical records system.

### Statistical analysis

Medians and interquartile ranges (IQRs) were used to describe continuous variables. Statistical tests for differences in continuous variables were performed with the Kruskal-Wallis test. Dunn test with Holm adjustment was applied to assess pairwise comparisons after the Kruskal-Wallis test.^[13]^ The Spearman rank correlation coefficient was used to explore the association between ISS and GCS. The χ^2^ test and Fisher exact tests were used for differences in proportions. A χ^2^ test of goodness of fit was performed to test the null hypothesis of equal numbers of cases by year. For some contingency tables (age group by year, discharge destination, injury mechanism by year, and injury mechanism by age), *P*-values for the Fisher exact test were calculated by Monte Carlo simulation with 2000 replicates.^[14]^

Data management, analysis, and visualization were performed with R version 4.4.2,^[14]^ the RStudio Integrated Development Environment 2024.12.0 Build 467,^[15]^ and 3 R packages: R Commander version 2.9–5,^[16,17]^ dunn.test,^[18]^ and ggpubr.^[19]^

## Results

### General characteristics of patients

General characteristics of patients are presented in Table 1. The number of cases in each calendar year was similar except for a lower number in 2020 (*P* < 0.001). The overall median age was 39 years (IQR: 25–58), and there were no differences (*P* = 0.28) between calendar years. Most cases (391; 41.3%) were young adults (20–39 years), and age group distributions were similar in all calendar years (*P* = 0.25). The majority of patients were men (764; 80.7%), and distribution by sex did not change over the course of the four-year study (*P* = 0.19). The overall median injury severity score (ISS) was 17 (IQR: 13–22). Data suggest differences in ISS between years 2019 and 2020, 2019 and 2022, and 2021 and 2022. The median Glasgow Coma Scale (GCS) remained low at 7 (IQR: 6–11) (*P* = 0.51, no differences detected). The overall median length of stay (LOS) was 20 days (IQR: 10–46.8) and remained consistent across the years (*P* = 0.24). There was a poor correlation between ISS and GCS (rho: 0.07; *P* = 0.03).

### Characteristics of patients and injuries by sex

**Figure 1.**
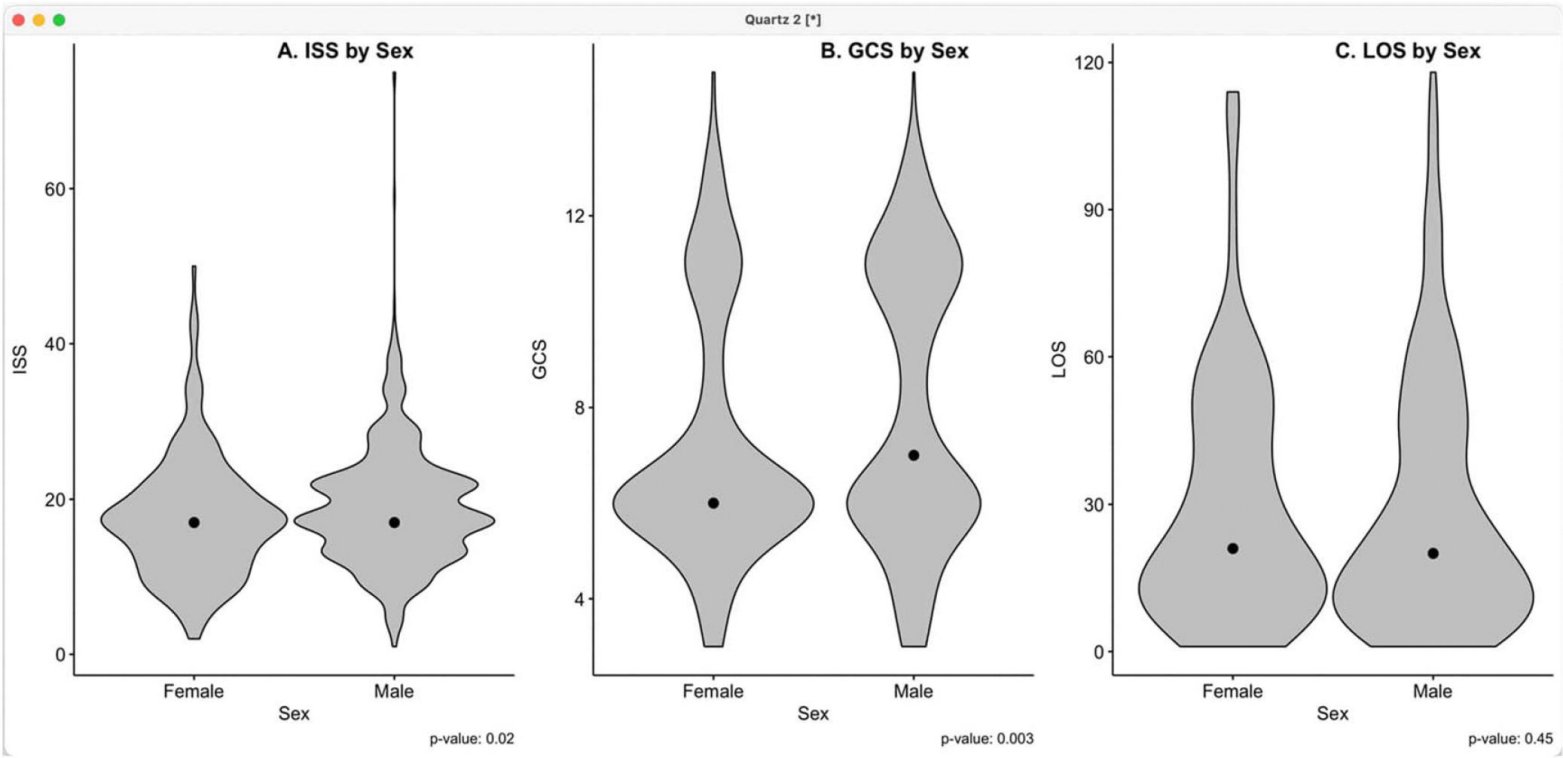
Violin plots for Injury severity score (A), Glasgow Coma score (B), and Length of Stay (C) by sex.

Table 2 shows the characteristics of TBI patients by sex. The median age in men was lower (38 y; IQR:26–55) than in females (47 y; IQR:24–72) (*P* = 0.006). The median ISS was similar for males and females, but the violin plots (Fig. 1) show 2 modes (scores 17 and 22) in the distribution for males, versus a single mode (score 17) in the distribution for females. This is compatible with slightly more severe injury in males (*P* = 0.02). The median GCS is slightly lower for females. Furthermore, IQRs and violin plots (Fig. 1) show a spread towards higher values in males (*P* = 0.003). These findings are compatible with more severe TBI in females. There was no difference between sexes in median length of stay (*P* = 0.24).

### Mechanisms of injury

There was no major difference in the distribution of mechanisms of injury by year (*P* = 0.14) (Table 3). Overall, motorcycle injuries (215; 23.0%) and motor vehicle collisions (214; 22.9%) were the leading mechanisms, followed by falls (186; 19.9%) and pedestrian injuries (181; 19.4%). Motorcycle injuries increased after 2019, but injuries associated with motor vehicle collisions decreased in 2020. Bicycle injuries were the least common cause of TBI (18; 1.9%) in all 4 years.

Table 4 shows differences in the distribution of injury mechanisms by age group (*P* < 0.001). The occurrence of falls increased with age. Motorcycle injuries predominantly affected younger age groups (0–39 years), with a very small number of cases after 60 years. The occurrence of motor vehicle collisions was similar in all age groups. Gun-related injuries primarily affected younger age groups (20–39 years), and no cases were reported in those 60 years or older. Data suggest pedestrian injuries increased with age during the period under study.

The proportion of TBI cases attributed to motor vehicle collisions was higher in females (73; 40.6%) compared with males (141; 18.7%), while the proportion of those attributed to motorcycles was higher for males (198; 26.3%) compared with females (17; 9.4%).

The ISS was lower in injuries caused by guns (median ISS: 14, IQR: 13–19) compared with motorcycles (ISS: 19; IQR: 14–22) (*P* = 0.01) and pedestrian (ISS: 17; IQR: 14–22) (*P* = 0.05) injuries. The GCS was lower in injuries caused by falls (GCS: 6; IQR: 6–11) when compared with guns (GCS: 11; IQR: 7–11) (*P* < 0.001) and in injuries caused by motorcycle collisions (GCS: 7; IQR: 6–11) compared with guns (*P* = 0.009). Guns were associated with higher GCS than motor vehicle collisions (GCS: 5; IQR: 6–11) (*P* < 0.001), and pedestrian injuries (GCS: 7; IQR: 6–11) (*P* = 0.01).

### Discharge disposition and in-hospital mortality

Most cases (684; 72.8%) were discharged home or to self-care (Table 5), but there were differences in disposition by year (*P* < 0.001). This proportion was lower in 2022, the same year when the proportion of patients discharged to inpatient rehabilitation was highest (*P* < 0.001). A total of 170 patients (18.1%) died in the THPR, and the mortality was similar in all 4 years.

Patient disposition showed differences by sex (*P* < 0.001). In-hospital mortality was higher (*P* < 0.001) for males (155; 20.4%) compared with females (15; 8.2%). Conversely, discharge home was higher (*P* < 0.001) among females (154; 84.6%) compared with males (530; 69.9%) (Fig. 1).

## Discussion

The most important findings of the present study were (1) the number of cases per year was similar except for a reduction in the year of the lockdown (2020) due to the pandemic, (2) most cases were men with severe injuries (low GCS; high ISS, prolonged LOS), (3) TBI was most frequent in younger men, (4) the most frequent mechanism of injury was motor vehicle and motorcycle collisions, while falls were more frequent in older adults, and (5) the majority of patients were discharged home and in-hospital mortality was high.

### Characteristics of patients and injuries

The number of TBI patients admitted to the THPR per year was similar throughout the study period, except for a decrease in 2020. This reduction coincided with the lockdown due to the COVID-19 pandemic. Others have reported a decrease in visits to a trauma unit during the pandemic, attributable to fewer motor vehicle injuries, occupational injuries, and sports participation.^[20–22]^ A decrease in TBI cases attributed to motor vehicle collisions in 2020 was also found in this study, though there were no major changes detected in mechanisms of injury.

The consistency in the number of cases during the other 3 years contrasts with a reported increase in incidence rates of TBI in the Caribbean from 1990 to 2021.^[4]^ The difference between the 2 studies may be related to the admission criteria to the THPR. The overall median ISS of 17 for TBI patients in the THPR is compatible with patients having additional major injuries at the time of admission.

Patients admitted with a TBI had low GCSs. The median GCS for patients in this study was 7, which is lower than in other reports. Mild TBIs with GCS of 13 and higher are more frequent in other countries, such as China,^[23]^ the Middle East,^[24]^ New Zealand,^[25]^ and United States.^[26]^

The predominance of men in our patient population is similar to that of other reports.^[25–27]^ Men consistently show a higher incidence of TBI when compared with females until the age of 70–79.^[4]^ This may be explained, at least partially, by the higher participation of men in high-risk activities.^[28]^ Our data also showed a higher number of TBIs in younger populations, which is like the findings in other countries.^[29]^ A possible explanation for this finding is a high level of social activity and the prevalence of higher-risk driving behaviors among young males.^[29]^

However, our data did not support a bimodal age distribution for TBIs. Large epidemiologic studies have shown a higher incidence of TBI in ages below 25 and above 75 years old.^[30]^ Others have observed an increasing incidence of TBI in the elderly.^[29,31]^ Globally, falls have been established as the leading cause of TBI.^[5,32]^ Yet, fall-related TBI patients in Puerto Rico are more likely to be admitted to other hospitals not specializing in trauma with complex associated injuries.

### Mechanisms of Injury

The most common mechanism of injury for TBI was motorcycle accidents, followed closely by motor vehicle collisions, falls, and pedestrian injuries. Factors that may have contributed to the predominance of motor vehicle injuries in the present study include a deficient road infrastructure,^[33]^ alcohol consumption,^[34]^ and speeding.^[35]^ Regarding the latter, Caetano et al^[36]^ conducted a survey of 1510 residents in the capital city and found a self-reported driving under the influence rate of about 1 in every 5 men and almost 1 in every 10 women.

Like the findings in the present study, TBI is also linked to the frequency of road injuries in other countries.^[5]^ According to Razaak et al,^[37]^ in low-income and middle-income countries, road injuries are the primary cause of TBI and result in poorer outcomes. Although road traffic accidents are a major cause of TBI in developed countries, falls have become the predominant mechanism of injury in these countries.^[5]^ Factors identified as important contributors to these findings include a rapid increase in motor vehicle ownership, insufficient road infrastructure, high rates of driving while intoxicated, limited policing of traffic, and inconsistent access to adequate trauma services, including post-acute care. To address this problem, several preventive strategies have been recommended, including reducing drunk driving via the development of public health campaigns to increase awareness, increasing legislation and law enforcement of appropriate seat belt and helmet use, and education on child restraints and boosters.^[38]^

### Discharge disposition and in-hospital mortality

In this study, discharge to home or self-care after a TBI was high. However, there was a trend toward higher discharge to an inpatient rehabilitation facility (IRF) in 2022. This coincides with the addition of a full-time brain injury rehabilitation specialist to the faculty of the THPR in September 2021. The addition of a physiatry consultation service in the trauma setting has been linked to shorter length of stay when consulted earlier.^[39]^ There is growing evidence that supports high-quality inpatient rehabilitation after moderate to severe TBI.^[40]^ Most patients with persistent disorder of consciousness, even at the time of admission to an inpatient rehabilitation facility (IRF), recover consciousness during rehabilitation.^[41]^ Yet, despite these positive findings, there has been a decreasing trend in admissions of severely disabled TBI patients to IRFs which contradict established guidelines for this population.^[42]^

Length of stay has been shown to be higher in patients who are deceased at 1 year following injury.^[43]^ In-hospital mortality in our study was high, particularly in males, but similar to mortality reported in other studies^[23,44]^ and did not change throughout the 4-year period. It is important to note that overall case fatality after TBI may be higher because 80% of deaths, particularly traumatic deaths, occur outside of health care settings. Age, GCS score, and ISS have been linked to TBI-related mortality.^[23,45,46]^

Interestingly, in-hospital mortality after TBI has been reported to show a trend toward a reduction^[44,47]^ linked to, among other factors, training and availability of surgeons, improved critical care, and training programs in the management of trauma for health care professionals.^[48]^

### Study limitations

The present study is limited to the TBI cases admitted to a level 2 trauma center and may not represent all cases of TBI in Puerto Rico. It is likely that mild and some moderate cases may be treated in other health care facilities and that only more severe cases are admitted to the THPR. Receiver operator characteristics (sensitivity and specificity) of the initial case definition based on diagnostic codes may not be constant among different trauma centers. This may add some degree of uncertainty to the final detection of TBI cases. However, the definition is supported by the US National Center for Injury Prevention and Control and the US National Center for Health Statistics, and it is widely used for research and surveillance. Finally, we based our study on secondary analysis of health care data, which might be subject to reliability issues.

## Conclusions

TBI care is a service in high demand in Puerto Rico, with a high burden and severity of injury among young men. The next step should include a detailed characterization of TBI in other hospitals in Puerto Rico and on strengthening rehabilitation services in health systems in the island. Comprehensive preventive strategies are required to address key mechanisms of TBI.

## Figures and Tables

**Table 1:** General characteristics of patients by calendar year.

	Year					
	
Variable	2019	2020	2021	2022	Total	*P*

No. cases	258	177	248	264	947	< 0.001
Median age, y (interquartile range)	38.5 (26.0–58.8)	41.0 (25.0–63.0)	36.5 (24.0–52.0)	39.0 (27.0–58.3)	39.0 (25.0–58.0)	0.28
Age groups, n (%)						
0–19	20 (7.8)	18 (10.2)	31 (12.5)	27 (10.2)	96 (10.1)	0.25
20–39	115 (44.6)	66 (37.3)	101 (40.7)	109 (41.3)	391 (41.3)	—
40–59	62 (24.0)	42 (23.7)	70 (28.2)	66 (25.0)	240 (25.3)	—
60–79	43 (16.7)	42 (23.7)	32 (12.9)	44 (16.7)	161 (17.0)	—
80+	18 (7.0)	9 (5.1)	14 (5.6)	18 (6.8)	59 (6.2)	—
Male gender, n (%)	209 (81.0)	149 (84.2)	204 (82.3)	202 (76.5)	764 (80.7)	0.19
Median Injury Severity Score* (Interquartile range	19 (14–24)	17 (13–22)	18 (14–22)	17 (13–22)	17 (13–22)	< 0.001
Median Glasgow Coma Scal(interquartile range) no data for 5 records	7 (6–11)	7 (6–11)	6 (6–1)	6 (6–11)	7 (6–11)	0.51
Median length of stay (d, interquartile range) no data for 1 record	18.5 (10.0–38.8)	21.0 (9.0–45.0)	21.0 (11.0–47.0)	21.0 (11.5–47.0)	20.0 (10.0–46.8)	0.24

* Dunn test *P*-values for differences in ISS: 2019 versus 2020—0.02; 2019 versus 2022: 0.001; 2021 versus 2022—0.01.

**Table 2:** General characteristics by sex.

	Sex			
		
Variable	Female	Male	Total	*P*

No. cases	183 (19.3)	764 (80.7)	947 (100.0)	< 0.001
Median age y (interquartile range)	47.0 (24.0–72.0)	38.0 (25.8–55.0)	39.0 (25.0–58.0)	0.006
Age groups (%)				
0–19	23 (12.6)	73 (9.6)	96 (10.1)	< 0.001
20–39	56 (30.6)	335 (43.8)	391 (41.3)	—
40–59	36 (19.7)	204 (26.7)	240 (25.3)	—
60–79	41 (22.4)	120 (15.7)	161 (17.0)	—
80+	27 (14.4)	32 (4.2)	59 (6.2)	—
Median Injury Severity Score (interquartile range	17 (13–22)	17 (13.8–22)	17 (13–22)	< 0.02
Median Glasgow Coma Scale (interquartile range)	6 (6–9)	7 (6–11)	7 (6–11)	0.003
Median length of stay (d, interquartile range)	21 (11–47)	20 (10–45.0)	20.0 (10.0–46.8)	0.24

**Table 3:** Injury mechanism by year.

	Year					
	
Variable	2019	2020	2021	2022	Total	*P*

Injury mechanism (%)						
Bicycle	7 (2.8)	4 (2.3)	4 (1.6)	3 (1.1)	18 (1.9)	0.14
Fall	43 (17.4)	42 (24.0)	45 (18.2)	56 (21.2)	186 (19.9)	—
Guns	14 (5.7)	8 (4.6)	8 (3.2)	9 (3.4)	39 (4.2)	—
Motorcycle	40 (16.2)	47 (26.9)	64 (25.9)	64 (24.2)	215 (23.0)	—
Motor vehicle collisions	63 (25.5)	29 (16.6)	61 (24.7)	61 (23.1)	214 (22.9)	—
Pedestrian	57 (23.1)	33 (18.9)	47 (19.0)	44 (16.7)	181 (19.4)	—
Other	23 (9.3)	12 (6.9)	18 (7.3)	27 (10.2)	80 (8.6)	—
Total*	247 (100.0)	175 (100.0)	247 (100.0)	264 (100.0)	933 (100.0)	—

* Fourteen records had no data for injury mechanism.

**Table 4:** Injury mechanism by age.

	Age group						
	
Variable	0–19	20–39	40–59	60–79	80+	Total	*P*

Injury mechanism (%)							
Bicycle	1 (1.1)	5 (1.3)	11 (4.7)	1 (0.6)	0 (0.0)	18 (1.9)	< 0.001
Fall	14 (14.7)	18 (4.7)	49 (21.0)	71 (44.7)	34 (57.6)	186 (19.9)	—
Guns	6 (6.3)	27 (7.0)	6 (2.6)	0 (0.0)	0 (0.0)	39 (4.2)	—
Motorcycle	28 (29.5)	147 (38.0)	38 (16.3)	2 (1.3)	0 (0.0)	215 (23.0)	—
Motor vehicle collisions	28 (29.5)	92 (23.8)	55 (23.6)	25 (15.7)	14 (23.7)	214 (22.9)	—
Pedestrian	10 (10.5)	57 (14.7)	56 (24.0)	48 (30.2)	10 (16.9)	181 (19.4)	—
Other	8 (8.4)	41 (10.6)	18 (7.7)	12 (7.5)	1 (1.7)	80 (8.6)	—
Total*	95 (100.0)	387 (100.0)	233 (100.0)	159 (100.0)	59 (100.0)	933 (100.0)	—

* Fourteen records had no data for injury mechanism.

**Table 5:** Disposition by year.

	Year					
	
	2019	2020	2021	2022	Total	*P*

Discharged to (%)						
Home or self-care (routine discharge)	201 (77.9)	131 (74.4)	186 (75.6)	166 (63.8)	684 (72.8)	< 0.001
Morgue	47 (18.2)	37 (21.0)	44 (17.9)	42 (16.2)	170 (18.1)	—
Rehab (inpatient)	4 (1.6)	4 (2.3)	6 (2.4)	36 (13.8)	50 (5.3)	—
Other	6 (2.3)	4 (2.3)	10 (4.1)	16 (6.2)	36 (3.8)	—
Total*	258 (100.0)	176 (100.0)	246 (100.0)	260 (100.0)	940 (100.0)	—

* Seven records had no data for disposition.
